# Insight into convergence of genetic and epigenetic mechanisms in reproductive organs in Arabidopsis

**DOI:** 10.3389/fpls.2026.1993320

**Published:** 2026-10-15

**Authors:** Karolina Zolkiewicz, Damian Gruszka

**Affiliations:** Faculty of Natural Sciences, Institute of Biology, Biotechnology and Environmental Protection, University of Silesia, Katowice, Poland

**Keywords:** Arabidopsis, DNA methylation, epigenetics, reproductive development, small interfering RNA, transcription factors

## Abstract

Mechanisms of epigenetic regulation of gene expression are inter-connected to ensure maintenance of DNA methylation. However, regulation of DNA methylation which allows new epigenetic patterns in various tissues during plant reproduction has remained incompletely understood. Recent reports have provided new and interesting discoveries in the field of epigenetic mechanisms regulating development of reproductive organs in the model plant species – *Arabidopsis thaliana*. As a consequence, an important, novel insight was gained into the role of several regulators, including GENETICS DETERMINES EPIGENETICS1 (GDE1) protein, as well as REPRODUCTIVE MERISTEM (REM) and REPRODUCTIVE MERISTEM INSTRUCTS METHYLATION (RIMs) transcription factors in the emergence of differential DNA methylation and small interfering RNA (siRNA) expression patterns in ovules and anthers during reproductive development in this model species. In this emerging view, genetic (transcription factors and recognized DNA motifs) and epigenetic (siRNA and DNA methylation) mechanisms converge to enable organ-specific DNA methylation during reproductive plant development.

## 1 Introduction

Gene expression in Eukaryotes is regulated at multiple levels. Initiation of transcription and role of transcription factors (TFs), which bind to gene promoters and cis-regulatory elements (CREs), constitute a foundation of this process and are crucial for proper regulation of plant development and responses to environmental cues. Moreover, the interactions between TFs and CREs are currently becoming a promising target for biotechnological improvement of crop species (Schmitz et al., 2022; Navratilova et al., 2026). Importantly, transition from the vegetative to the reproductive stage is a fundamental process during plant development. This process is regulated genetically and epigenetically and depends on mechanisms modulating plant development and the responses to the environmental cues (Liu et al., 2024; Navratilova et al., 2026).

As far as the epigenetic mechanisms are concerned, they encompass two separate and opposite processes: the maintenance of chromatin modification patterns during cell division and ability to generate novel, diverse epigenetic patterns during plant development (Calarco et al., 2012). DNA methylation is known to be maintained through the positive inter-connections with other repressive epigenetic mechanisms (histone methylation and non-coding RNAs). The inter-connections enable silencing of genes and transposons and maintain genome stability to control physiology of various organisms during their development and adaptation to environmental conditions (Slotkin and Martienssen, 2007; He et al., 2009; Law and Jacobsen, 2010; Xu and Law, 2024; Wu et al., 2025). Until recently, the mechanisms which allow establishing new and diverse epigenetic patterns, in the DNA methylation-dependent manner, in various tissues during plant development and reproduction have remained largely unknown (Martins and Law, 2023; Xu and Law, 2024). However, the recent articles have provided new and interesting discoveries in the field of epigenetic mechanisms regulating development of reproductive organs in the model plant species – *Arabidopsis thaliana* (Wu et al., 2025; Xu et al., 2025). In Arabidopsis, the pattern of DNA methylation, in three different sequence contexts: CG, CHG or CHH (where H is A, T or C), is established by the RNA-directed DNA methylation (RdDM) mechanism in which plant-specific RNA polymerases (Pol-IV and Pol-V) transcribe loci encoding 25–40-nucleotide-long small interfering RNAs (siRNAs) and long non-coding RNAs, respectively, which regulate DNA methylation (Chan et al., 2004; Herr et al., 2005; Onodera et al., 2005; Pontier et al., 2005; Wierzbicki et al., 2008; Matzke and Mosher, 2014; Rymen et al., 2020). Types of plant siRNAs (determined by the siRNA precursors), stages of their biogenesis, and roles of the major enzymes regulating this process, e.g. RNA-Dependent RNA polymerases (RDRs), Dicer-like (DCL) proteins, and Hua Enhancer 1 (HEN1) methylase were described. Functions of the Argonaute (AGO) effector proteins which are components of the RNA-induced silencing complex (RISC) involved in directing mature sRNA to its target mRNA, as well as Domains Rearranged Methyltransferases (DRMs) which mediate the siRNA-dependent epigenetic regulation of target gene expression were also characterized (reviewed by Guleria et al., 2011; Tang et al., 2022; Tan et al., 2024).

In Arabidopsis, the molecular RdDM machinery is directed to various loci (including *siRNAs*) by a group of CLASSY (CLSY1-4) SWI2/SNF2-like chromatin remodelling factors which function as tissue- and locus-specific regulators of DNA methylation. Tissue-specific expression enables the CLSY chromatin remodelling factors to generate epigenetic diversity during plant development (Zhou et al., 2018; Yang et al., 2018; Zhou et al., 2022). It is known that CLSY1 and CLSY2 play a predominant role in the siRNA expression in leaves, whereas CLSY3 and CLSY4 are highly expressed and active at specific siRNA loci - *siren* (small-interfering RNA in the endosperm) and *hyperTE* (hypermethylated transposable element) in ovules and tapetum cells within anthers, respectively (Zhou et al., 2022; Wu et al., 2025). Interestingly, it was reported that in contrast to loci regulated by CLSY1 and CLSY2, expression of the *siren* loci controlled by CLSY3 and CLSY4 is not dependent on the non-CG DNA methylation and proceeds independently of the histone H3 lysine-9 (H3K9) methylation (Liu et al., 2011; Law et al., 2011, 2013; Zhou et al., 2018, 2022). Therefore, it was inferred that during the evolution the CLSY proteins developed diverse strategies of recruitment to target *siRNA* loci: some are dependent on the epigenetic features, whereas others are based on the sequence-specific genetic information. These strategies enable flexible and tissue-specific control of the siRNA expression (Wu et al., 2025). It indicated that these *siRNA* loci are regulated by a distinct mechanism than other RdDM target loci, and their expression is dependent on a conserved DNA motifs which influence the epigenetic process (Zhou et al., 2022; Wu et al., 2025; Xu et al., 2025). Within the reproductive organs of Arabidopsis, the RdDM machinery is directed to various loci by CLASSY3 what allows for establishing various epigenomes. However, the exact mechanism of emergence of these distinct epigenetic patterns has remained incompletely understood until recently (Wu et al., 2025; Xu et al., 2025).

## 2 The role of novel REM and RIM transcription factors in the emergence of distinct DNA methylation and siRNA expression patterns regulating plant reproductive development

A recent report by Wu et al. (2025) indicated that several REPRODUCTIVE MERISTEM (REM) TFs, which belong to a large and swiftly evolving family expressed mainly in the reproductive tissues (Mantegazza et al., 2014), recognize the DNA motifs and together with the GENETICS DETERMINES EPIGENETICS1 (GDE1) protein recruit the RNA Pol-IV transcription complexes to initiate production of siRNAs which guide DNA methylation to specific loci in female reproductive tissues (ovules) in Arabidopsis. This important discovery established a direct link between the above-mentioned genetic elements (DNA motifs, TFs), the spatial regulation of siRNA production, and DNA methylation, providing new insights into the genetic control of epigenetic patterning (Wu et al., 2025; Xu et al., 2025). It was reported that GDE1 was highly enriched at both CLSY3 and CLSY4 binding sites, and the *GDE1* gene shared with the *CLSY3* and *CLSY4* genes a common and unique expression pattern in flower tissues (Zhou et al., 2022; Wu et al., 2025). These results suggested that GDE1 cooperates with the CLSY3 and CLSY4 proteins to modulate the siRNA production. Moreover, analysis of the CLSY3/CLSY4-dependent siRNA expression profiles (in ovules of the *gde1–1* mutant) indicated that the GDE1 and the CLSY3/CLSY4 proteins showed overlapping and differential siRNA modulation function. Interestingly, it was also concluded that GDE1 plays a crucial role in recruiting the CLSY3/CLSY4–RNA polymerase IV (Pol-IV) complex to produce siRNAs in a subgroup of the CLSY3/CLSY4-dependent *siRNA* loci, and that in the absence of GDE1 these complexes are redistributed to other loci. Noteworthy, any of the *gde1-1*, *clsy3 clsy4* and *pol iv* mutants did not display clear developmental phenotypes (Wu et al., 2025). Moreover, these analyses allowed for emergence of an interesting view in the genome-wide perspective: GDE1 primarily regulates siRNA production in euchromatic regions, whereas it competes with the Pol-IV recruitment to heterochromatic regions. Further experiments indicated that GDE1 forms a complex with CLSY3 and Pol IV and identified other interacting proteins from the REM family of TFs in Arabidopsis - VIVIPAROUS1/ABI3-LIKE (VAL), VERDANDI (VDD), REM12, and REM13 (and to a lesser extent REM8, REM19, and REM22) - which together with GDE1 recognize the specific CLSY3/CLSY4-dependent *siRNA* loci and recruit the Pol-IV transcription complexes. This recruitment results in the production of siRNAs which guide the DNA methylation to specific loci in ovules (**Figure 1**). These REM TFs were predicted to bind at least 28% of the CLSY3/CLSY4-dependent *siRNA* loci. As far as tissue-specific activity is concerned, it was reported that the REM–GDE1 complexes may recruit CLSY3/CLSY4 to the *siren* loci in ovules to regulate the siRNA expression and (CHH- and CHG-type) DNA methylation (**Figure 1**; **Table 1**). This dependency seems mutual, as it was also verified that both GDE1 and CLSY3/CLSY4 participate in stabilization of the REM TFs to the *siren* loci. Another important conclusion of this study indicated that the REM TFs regulate the siRNA expression independently of the H3K9 methylation (Wu et al., 2025). In previous studies, the H3K9 methylation was reported to be involved in the recruitment of Pol-IV transcription complexes to the *siRNA* loci (Law et al., 2013; Zhou et al., 2018). Additionally, it was also reported in the study by Wu et al. (2025) that in the absence of GDE1, the Pol-IV transcription complexes are recruited to loci bound by an alternative TF - REM8. This observation validated the role of the REM TFs and the GDE1 protein as positional regulators of the epigenetic modulation (Wu et al., 2025). It was suggested that the REM8 TF may interact with other (yet not identified) factors to recruit the CLSY3/CLSY4–Pol IV complex to a subset of the *siRNA* loci. Noteworthy, it was also reported that the *GDE1* gene is highly expressed in the tapetum cells and involved in modulation of the siRNA expression in anthers. However, this regulation seems to be independent of the tested REM TFs. Thus, it was concluded that GDE1 is involved in the regulation of siRNA production in anthers, but through a different mechanism than the one reported in ovules and unknown recruiters or transcription factors are presumably involved (Wu et al., 2025) (**Figure 1**).

**Figure 1:**
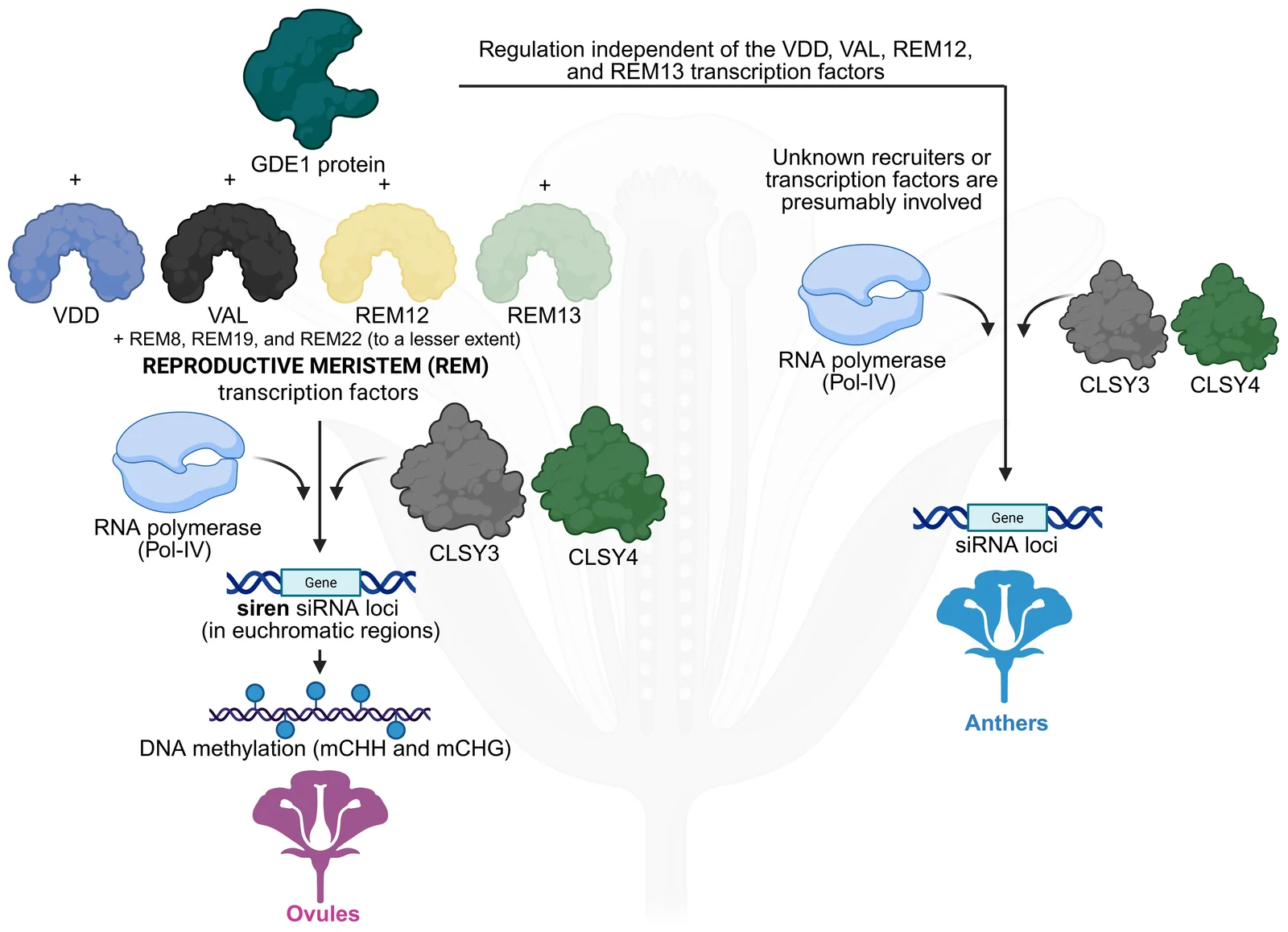
Role of the GDE1 protein and REPRODUCTIVE MERISTEM (REM) transcription factors in the CLSY3/CLSY4– and RNA polymerase IV (Pol-IV)-dependent regulation of production of siRNAs which guide DNA methylation in Arabidopsis reproductive tissues. Details are given in the text. Created with BioRender.com.

**Table 1:** Characteristics of the REM/RIM-type transcription factors.

REM/RIM-type TF	Gene ID	Reproductive tissue	Target loci	CLSY- dependence	Methylation context	Mutant phenotype	References
VAL/REM11/RIM11	AT5G60140	ovules	*siren*	yes	CHH- and CHG-type	strong female gametophytic defects, reduced fertility	Mendes et al., 2016; Wu et al., 2025; Xu et al., 2025
VDD/REM20	AT5G18000	ovules	*siren*	yes	CHH- and CHG-type	strong female gametophytic defects	Mendes et al., 2016; Wu et al., 2025
REM12/RIM12	AT5G60130	ovules	*siren*	yes	CHH- and CHG-type	normal viability and fertility	Wu et al., 2025; Xu et al., 2025
REM13	AT3G46770	ovules	*siren*	yes	CHH- and CHG-type	delay in flowering time, normal viability and fertility	Manrique et al., 2023; Wu et al., 2025
RIM16/REM16	AT3G53310	anthers (mainly)	*HyperTE*	yes	mainly CHH-type	delay of floral transition, normal viability and fertility	Yu et al., 2020; Xu et al., 2025
RIM22/REM22	AT3G17010	anthers (mainly)	*HyperTE*	yes	mainly CHH-type	decrease in ovule number	Gomez et al., 2018; Xu et al., 2025
RIM46/REM46	AT5G60142	ovules	*siren*	yes	mainly CHH-type	early flowering phenotype, normal viability and fertility	Manrique et al., 2023; Xu et al., 2025

Another recent study has broadened the emerging view of the convergence of genetic and epigenetic mechanisms through description of several REM TFs which were functionally characterized using forward- and reverse-genetics approaches. These TFs belong to the above-described REM family and were designated as REM INSTRUCTS METHYLATION (RIMs), as they are required for the differential, CLASSY3-mediated DNA methylation (mainly the CHH-type methylation) at distinct *siRNA* loci specific to anthers and ovules in Arabidopsis (Xu et al., 2025). In terms of the *siRNA* loci, this study was focused on the *HyperTE* and *siren* loci that are predominant among siRNAs expressed in anthers and ovules, respectively (Grover et al., 2020; Zhou et al., 2022; Xu et al., 2025). The role of RIMs was confirmed through disruption of their DNA-binding domains or alterations in their recognized DNA motifs. Both changes resulted in a blockage of RdDM at the *HyperTE* and *siren* loci. It was also reported that altered expression of one of the RIMs led to siRNA production in anthers from loci which are normally expressed in ovules. Importantly, it was reported that all RIMs regulate CLASSY3-dependent RdDM, however, mutations in two RIMs (*rim16* or *rim22*) affect mainly the *HyperTE* loci in anthers, whereas a deletion of three tandem genes (*rim11, 12, 46*), developed through the CRISPR/Cas9 approach, selectively affects the *siren* loci in ovules. Interestingly, the *rim16* mutation decreased methylation at four out of five *HyperTE* loci, but not at the *siren* locus. Similarly, the *rim22* mutation influenced siRNAs at ca. two-third (434/715) of the *HyperTE* loci, but only one *siren* locus. It indicated that RIM16 and RIM22 are slightly less tissue-specific than the other RIMs and play a role in regulating mainly the *HyperTE* loci in anthers. Interestingly, it was reported that RIM16 and RIM22 regulate some *HyperTE* loci independently, while other *HyperTE* loci in a redundant manner. However, it was also suggested that additional factors are required to regulate DNA methylation at these loci. On the contrary, in the above-mentioned triple CRISPR mutant (*rim11, 12, 46*) DNA methylation was reduced at the *siren* loci, but none of the *HyperTE* loci (**Table 1**). Noteworthy, no redundancy was reported between the RIM16 and RIM22 pair of TFs and the RIM11, RIM12, and RIM46 TFs (Xu et al., 2025). An illustrative model of regulation of the above-described processes in the different reproductive organs is presented in **Figure 2**. In this way, it was demonstrated that the disruptions of different RIMs’ functions prevent the proper RdDM progress which is of key importance for unique DNA methylation patterns being important for anther and ovule development. Importantly, it was reported that the *RIM16* and *RIM22* genes showed higher expression in anthers than in ovules, whereas the *RIM11*, *RIM12*, and *RIM46* genes exhibited an opposite expression pattern. Therefore, these distinct groups of transcription factors (RIM16, RIM22) and (RIM11, RIM12, RIM46) act in locus- and tissue-specific manner in regulating RdDM which influences expression of the majority of the *HyperTE* and *siren* loci in anthers and ovules, respectively (Xu et al., 2025) (**Figure 2**; **Table 1**).

**Figure 2:**
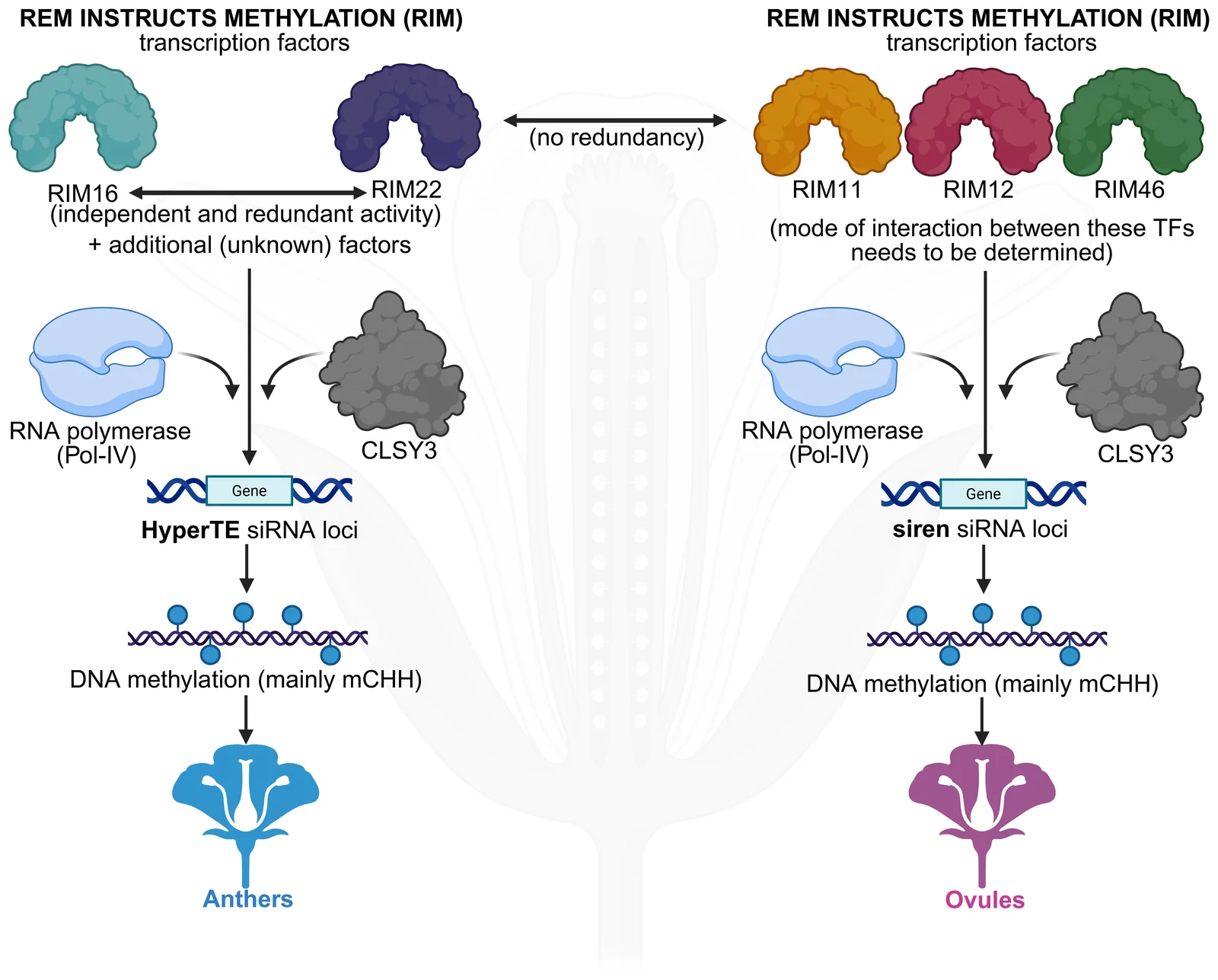
Role of the REPRODUCTIVE MERISTEM INSTRUCTS METHYLATION (RIM) transcription factors which are required for differential DNA methylation at distinct small interfering RNA (*siRNA*) loci in different organs during reproductive development in Arabidopsis. Details are given in the text. Created with BioRender.com.

## 3 Conclusions and perspectives

The research by Wu et al. (2025) provided the important, novel insight into the role of REM TFs in a direct association between sequence-specific TFs and the spatial regulation of the siRNA expression and DNA methylation in the reproductive tissues of Arabidopsis (**Figure 1**). In this model, the REM TFs in cooperation with the GDE1 protein recruit the CLSY3/CLSY4–Pol IV complexes to regulate the siRNA expression and subsequent DNA methylation at target loci in ovules. However, it should be kept in mind that the above-mentioned, various REM TFs may redundantly form different combinatory heterodimers to allow for broader recruitment at the *siRNA* loci and differential regulation of their expression, also in the CLSY3/CLSY4-independent manner (Wu et al., 2025). The study by Xu et al. (2025) characterized RIMs as crucial components of the RdDM process, with the particular emphasis on their specific role in development of distinct reproductive tissues. This role distinguishes RIMs from other RdDM components. It provided a very important and interesting insight into the profound role of genetic information and function of the RIM TFs in the spatial regulation of DNA methylation profile at distinct *siRNA* loci in reproductive tissues of Arabidopsis. In this emerging view, genetic (TFs and recognized DNA motifs) and epigenetic (siRNA and DNA methylation) mechanisms converge to enable organ-specific patterns of DNA methylation during reproductive development of the model plant species (**Figure 2**).

Although the recent studies provided the very important new insight into the crucial aspects of plant biology, some questions still await to be answered. Firstly, given the total number (46) of the REM TFs expressed primarily in reproductive tissues in Arabidopsis and the fact that additional factors are required to regulate RdDM at the analyzed loci, it needs to be determined if any other REM TFs play a role in this process in the organ-dependent manner and to what extent their roles are independent or redundant? The aforementioned studies (Wu et al., 2025; Xu et al., 2025) were performed in Arabidopsis being a model species for dicots. However, it would be very interesting to verify whether the above-mentioned mechanisms are conserved in monocot species, especially cereal crops? This issue needs to be clarified. However, some differences in this mechanism between dicots and monocots may exist, as it was reported that in contrast to Arabidopsis mutants defective in RdDM, which show normal fertility, mutants of cereal crops, such as maize (*Zea mays*) and rice (*Oryza sativa*), deficient in the RdDM process, exhibited abnormalities in reproduction (Erhard et al., 2009; Pal et al., 2024). In rice, the OsCLSY3 chromatin remodeler plays a key role during endosperm development, acting upstream in the siRNA pathway. It was shown that the *OsCLSY3* gene is preferentially expressed in endosperm in the RdDM-dependent manner, and regulated by transposable elements (TE)-derived siRNAs and the *siren* loci. Abnormalities in the OsCLSY3 function in transgenic rice lines caused defects in endosperm development, sterility, and a significant decline in expression of around 70% of all siRNAs. Interestingly, *osclsy3* knockout mutants did not display growth defects at the vegetative stages of development. However, the *OsCLSY3* overexpression resulted in larger grains, but with defective cellularization. Noteworthy, expression of the endosperm-specific *siren* loci and seed development-associated genes is also OsCLSY3-dependent (Pal et al., 2024). On the other hand, the above-mentioned *VDD* and *VAL* genes were reported to promote fertilization through controlling degeneration of synergid cell in Arabidopsis and loss-of-function mutations of these genes cause strong female gametophytic defects (Mendes et al., 2016). The lethality of the knockout mutants and the multitude of the REM TFs make genetic and functional analysis a challenging task (Wu et al., 2025). However, it was reported that expression of the *GAMETOPHYTIC FACTOR 2* (*GFA2*) gene which plays a role of key regulator of synergid cell death may rescue the phenotypic effects of these mutants (Mendes et al., 2016). Potentially, this approach may constitute a solution for the phenotypic defects issue. Another methodological (gain-of-function) approach has also been recently suggested (Wu et al., 2025): in this study the VDD TF was fused with the artificial zinc finger 108 (ZF) protein which target hundreds of binding sites in the Arabidopsis genome (Gallego-Bartolomé et al., 2019; Wang et al., 2023). In the VDD–ZF transgenic plants, siRNAs were produced *de novo* at many of the ZF fusion targeted regions in the ovule tissues, indicating the sufficiency of REM TFs to promote siRNA expression (Wu et al., 2025). Potentially, broadening the knowledge on these aspects may provide promising targets for biotechnological manipulations aimed at improvement of reproduction and yield of the crop species. However, further research on these aspects in the crop species is needed. It would be also interesting to verify to what extent the above-mentioned mechanisms are regulated by endogenous (phytohormones) and/or exogenous (environmental) factors, particularly stress conditions, taking into account that variations in epigenetic players, including CLSYs, may contribute to adaptation to diverse environmental conditions (Martins and Law, 2023; Pal et al., 2024).
